# Comparison Between Detection of Orthodontic‐Related Carious Lesions With Fluorescence or Visual Method: A Systematic Review

**DOI:** 10.1111/idh.70041

**Published:** 2026-03-19

**Authors:** Luciana Pereira da Silva, Fernanda Mafei Felix da Silva, Marcela Baraúna Magno, Daniele Masterson Tavares Pereira Ferreira, Amanda Cunha Regal de Castro, Maria Augusta Visconti, Lucianne Cople Maia, Aline de Almeida Neves

**Affiliations:** ^1^ Department of Pediatric Dentistry and Orthodontics, School of Dentistry Federal University of Rio de Janeiro Rio de Janeiro RJ Brazil; ^2^ Central Library of the Health Science Center, Health Science Center Federal University of Rio de Janeiro Rio de Janeiro RJ Brazil; ^3^ Department of Pathology and Oral Diagnosis, School of Dentistry Federal University of Rio de Janeiro Rio de Janeiro RJ Brazil

**Keywords:** diagnosis, fluorescence, orthodontics, photographs, QLF, tooth demineralization

## Abstract

**Objective:**

To pare the detection of carious lesions involving orthodontic appliances (O) with the fluorescent method (I) and visual clinical examination (R) in patients treated with fixed orthodontics (P).

**Methods:**

Five databases and grey literature were searched until January 2023. Clinical studies, comparing at least one digital fluorescent method (DFM) with visual clinical examination (VCE), in patients who completed treatment with fixed orthodontic appliances, were eligible. After extracting the data, the risk of bias and the certainty of the evidence were evaluated through the QUADAS‐2 and the GRADE approaches, respectively.

**Results:**

Five cross‐sectional studies were included in the final synthesis. The number of teeth evaluated ranged from 137 to 1653. Quantification of lesion severity by determining fluorescence loss was a great benefit compared to the qualitative data obtained by VCE. The average loss of fluorescence, measured by quantitative light‐induced fluorescence (QLF) method, in the lesions visually detected was 12.6%, in some areas greater than 15%. The correlation between DIAGNODent and VCE scores ranged from 0.40 to 0.71. The studies were judged at risk of bias with very low certainty of evidence on the comparison between DFMs and VCE.

**Conclusion:**

The detection of non‐cavitated carious lesions by DFMs showed a similar pattern to the VCE, although the former detected more lesions than the latter, which encourages the use of these technologies for early detection in patients submitted to fixed orthodontic treatment.

**Clinical Relevance:**

The clinical advantage of using the DFMs include the more objective diagnosis, and that measurements can be displayed to the patients and may, therefore, have a pedagogical value. In contrast, they are more time consuming clinically and pose an extra cost; however, it is believed that rapid technology innovation may reduce these costs.

## 1 Introduction

Orthodontic fixed appliances constitute a mechanical obstacle for biofilm removal, favouring thus, enamel demineralization and further caries development [1, 2]. Indeed, there is already evidence to show that the presence of orthodontic appliances directly influences the amount and quality of the oral microbiota [3, 4]. The incidence of white spot lesions (WSLs) in patients undergoing orthodontic treatment is around 38%–40% in the first six months of treatment [5], increasing to 43%–46% in a 12‐month period, supporting the idea that fixed appliances potentially become a risk factor for carious lesions [6, 7]. Studies have shown that approximately one third of patients undergoing orthodontic treatments with fixed appliances developed at least one WSLs [8, 9]. In such cases, early detection may be a challenge due to the presence of the bonded accessories [10].

Caries diagnosis is a clinical judgement, integrating detection and assessment of signs, such as the presence of lesions, to determine presence of the disease and define the course of treatment [11]. In the clinical routine, it can be performed through visual clinical examination (VCE), at various thresholds and stages of detection. Nowadays, a widespread index used is the International Caries Detection and Assessment System (ICDAS‐II) [12], aided by complementary exams such as radiographs, optical and electrical methods [13].

The use of digital fluorescent methods (DFMs) has been increasing [14], in order to assist early enamel caries diagnosis. In clinical practice, instruments such as QLF [15, 16], DIAGNOdent Pen [10, 17, 18], and Vista Proof [10], are frequently used for detection of early caries. The carious lesion can be detected by light‐induced fluorescence, when its value decreases at the lesion site [19]. Although intraoral cameras still have a significant cost [10], their advantages include no emission of ionising radiation, the possibility of storing standardised images throughout the consultations to provide an independent review of the images at different times, and the possibility of classifying the presence or absence of the lesion according to the intensity of the fluorescence [10].

Considering all these methods and their possible applicability in clinical practice, the present systematic review intended to answer the following focused question: Are DFM and visual clinical examination comparable in detecting early carious lesions related to fixed orthodontic appliances?

## 2 Methods

### 2.1 Protocol Registration

Registered in PROSPERO under the number CRD42018109453. The authors also followed the recommendations of the PRISMA statement [20].

### 2.2 Search Strategy

Searches were performed in the following electronic bibliographic data sources: PubMed, Scopus, Web of Science, The Cochrane Library, Lilacs, BBO, as well as in the grey literature (Google Scholar and Trip Database). Medical Subject Headings (MeSH) terms and keywords were used to identify published papers. The search strategy was appropriately modified for each database according to their syntax rules in Table 1. The search was carried out including studies published until January 2023. Hand search in the references list of the included papers was also performed. No date or language restriction was applied.

**TABLE 1:** Search strategy of each database.

Database	Search estrategy
Pubmed	#1 (Optical Imaging[Mesh] OR Fluorescenc* [tiab] OR Photography[Mesh] OR Photograph*[tiab]) #2 (Orthodontic*[Tiab] OR Orthodontics[Mesh] OR Orthodontic Brackets[Mesh] OR Bracket*[tiab]) #3 (Dental Caries[Mesh] OR Carie*[Tiab] or White Spot Dental[tiab] OR white spot[tiab] OR Demineralization Dental[tiab]) #1 AND #2 AND #3
Scopus	#1 TITLE‐ABS‐KEY (“optical imaging” OR “fluorescence imaging” OR fluorescence* OR photograph*) #2 (orthodontic* OR bracket* OR “brace orthodontic”) #3 (carie* OR “White spot” OR “demineralization dental”) #1 AND #2 AND #3
ISI Web of Science	#1 (“optical imaging” OR “fluorescence imaging” OR fluorescence* OR photograph*) #2 (orthodontic* OR bracket* OR “brace orthodontic”) #3 (carie* OR “White spot” OR “demineralization dental”) #1 AND #2 AND #3
The Cochrane Library	#1 MeSH descriptor: [Dental Caries] #2 (Carie* OR “White Spot”):ti, ab, kw #3 MeSH descriptor: [Orthodontic Brackets] #4 (Brackets Orthodontic* OR Bracket*):ti, ab, kw #5 MeSH descriptor: [Optical Imaging] #6 (“Fluorescence Imaging” OR “Imaging Autofluorescence” OR Photography):ti, ab, kw #7 #1 OR #2 #8 #3 OR #4 #9 #5 OR #6 #10 #7 AND #8 AND #9
Lilacs/BBO	(mh:(optical imaging)) OR (mh:(fluorescence)) OR (mh:(photography)) OR (tw:(fluorescence imaging)) OR (tw:(imaging autofluorescence)) OR (tw:(photograph*)) OR (tw:(photography dental)) OR (tw:(photography orthodontic*)) OR (tw:(photographies intraoral))) AND ((mh:(orthodontic*)) OR (tw:(orthodontic treatment)) OR (mh:(orthodontic brackets)) OR (tw:(brackets orthodontic*)) OR (tw:(bracket*)) OR (tw:(brace orthodontic*))) AND ((mh:(dental caries)) OR (tw:(carie*)) OR (tw:(white spot dental)) OR (tw:(white spot)) OR (tw:(demineralization dental)) AND (db:(“LILACS” OR “BBO”) AND type:(“article”))
Open Grey	(Carie*) AND (Bracket*) AND (Fluorescence*)

Two investigators performed the search independently (L.P.S. and F.M.F.S.), under the guidance of a librarian (D.M.). All titles, abstracts and full‐text manuscripts retrieved from database searches were screened, excluding irrelevant records. In the case of any inconsistency, a third author (L.C.M.) was consulted. All references were imported into a reference manager online software (EndNote Web; Thomson Reuters Inc., Philadelphia, PA, USA) and duplicated references were considered only once.

### 2.3 Eligibility Criteria Based on PIRO Strategy and Selection Criteria

The PIRO strategy consists of an acronym for (P) patient, (I) intervention, (R) standard reference and (O) outcomes. Clinical studies in patients treated with fixed orthodontics (P), in which the evaluation within a fluorescent method (I) was compared to the visual clinical examination (R), in order to detect carious lesions involving orthodontic appliances (O), were considered eligible.

The following exclusion criteria were considered: studies in which pathologies other than carious lesions, such as dental fluorosis or molar incisor hypomineralization (MIH) and stains of other origin (since fluorescent devices are sensitive to any stain, for example, caused by the accumulation of biofilm) were excluded from this systematic review.

### 2.4 Data Extraction

A data extraction spreadsheet was developed, and data were collected independently by two researchers (L.P.S. and F.M.F.S.). For each selected study, the following information was collected: author/country/publication year, patient age/number of teeth evaluated, treatment time, orthodontic appliances, methodological evaluation, repetition of measurements, fluorescent method, visual clinical method, and outcomes. The authors of the primary studies were contacted in case of lack of relevant data.

### 2.5 Risk of Bias Analysis and Methodological Quality of the Studies

The evaluation of methodological quality in the included studies was performed according to the quality assessment of diagnostic accuracy studies (QUADAS‐2) [21].

In domain 1, the risk of bias regarding ‘patient selection’ was assessed using questions such as: Did the study involve a consecutive or random sample of patients? Have case–control design and inappropriate exclusions been avoided? The applicability of domain 1 was concerned with whether patients included in the study and settings corresponded to the review question.

In domain 2, the risk of bias regarding the use or interpretation of the ‘index test’, was assessed through questions referring to DFMs, whether they were interpreted without knowledge of the results of the reference standard (VCE), and whether a threshold was used and/or pre‐specified. The interpretation of this domain considered the following aspects: if the index test was performed according to manufacturer's recommendations, if the evaluator was experienced and/or calibrated and if a previous surface cleaning of the examined tooth was performed. The applicability of this domain was concerned to whether the ‘index test’ methods differed from those defined in the review question.

In domain 3, the risk of bias regarding the use or interpretation of the ‘reference standard’ was assessed through questions referring to VCEs, whether they were able to correctly classify the target condition and if its results were interpreted without knowledge of the ‘test index’ (DFMs). The interpretation of this domain considered validated VCE methods as reference standards for carious lesions assessment. The applicability of this domain was concerned with whether the ‘reference standard’ defined a target condition different from that described in the review question.

The fourth domain, ‘flow and time’, was concerned to investigate whether the patient flow might have introduced bias, considering if there was an adequate interval between the ‘index test’ and ‘reference standard’ assessments, in this one, simultaneous to the other; if all patients received the same ‘reference standard’ and if the study analysis comprised all the patients recruited.

Risk of bias and concerns regarding applicability were rated as ‘low’, ‘high’, or ‘unclear’, as follows: if all questions for a domain were answered with ‘yes’, the study was classified at ‘low risk’ of bias; if any question was answered with ‘no’, it was classified at ‘high risk’ of bias; and an ‘unclear’ status was assigned when insufficient data was provided. Therefore, the ‘overall judgement’ of the risk of bias and applicability was defined using the following criteria: if the risk of bias and/or applicability was rated as ‘low’ on all domains, the study was judged as ‘low risk of bias’ or ‘low concern regarding applicability’; and if it was rated as ‘high’ or ‘unclear’ in one or more domains, the study was judged ‘at risk of bias’ or with ‘concerns regarding applicability’ [21].

### 2.6 Certainty of Evidence

GRADE (Grading of Recommendations Assessment, Development and Evaluation) was used to analyse the certainty of evidence [22, 23] and adapted to the subject of the present systematic review. When serious or extremely serious issues related to risk of bias, inconsistency, indirectness, inaccuracy, and publication bias are observed, the quality or certainty of evidence decreases by one or two points. Conversely, if there is a large or very large magnitude of an effect, a dose–response was observed, or if the effect of all plausible confounding factors is minimised or suggests a spurious effect, the quality of evidence tends to increase by two points. In this respect, the certainty of evidence in GRADE may range between very low, low, moderate, or high.

For the criterion ‘risk of bias’, it was considered a ‘not serious’ problem if all included studies presented low risk of bias and a ‘very serious problem’ if the included studies presented ‘high’ risk of bias. For the “inconsistency” criterion, it was considered a very serious problem if the studies included in the systematic review presented a large variation in the effect estimates between studies.

The indirectness was assessed when the pooled results partially addressed the issue of interest for revision in the population (evidence evaluated should include bands and brackets), fluorescent method (evidence should include two or more fluorescent methods) and visual clinical method (evidence should include two or more visual clinical methods used). If there was a limitation in one of these criteria (studies included only bands or brackets, or only one fluorescent or visual clinical method) the problem was judged to be ‘serious’; if there was a problem in two or three criteria (studies included only bands or brackets, and only one fluorescent and/or visual clinical method), the problem was judged to be ‘very serious. In the analysis of ‘imprecision’, a serious problem was considered if the total number of teeth evaluated was less than 300 [23].

The criterion ‘publication bias’ was judged to be ‘undetected’ since the search was done in white and grey databases, with no date or language limitation. The criterion ‘dose–response’ does not apply to the studies included in this systematic review and was classified in such a way as not to modify the final classification of the evidence. For the ‘magnitude effect’, it was considered with a very large magnitude effect if all studies included reported strong correlation (> 0.7 or < 0.7) [24] or high accuracy (> 0.9) [25].

## 3 Results

### 3.1 Literature Search

**FIGURE 1:**
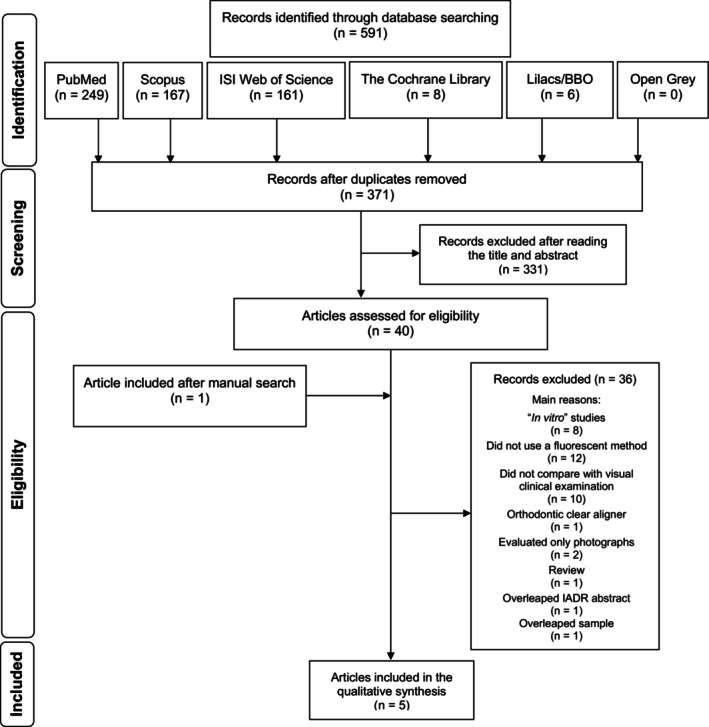
Flow diagram of the screening and selection process.

All of the 591 articles found were exported to EndNote Web software. All duplicates were removed, and 371 articles remained from which, after reading titles and abstracts, 331 were excluded. Forty full text articles were subsequently assessed for eligibility and 36 were excluded for the following reasons: “in vitro” studies (*n* = 8), did not use a fluorescent method (*n* = 12), did not use VCE as comparison (*n* = 10), used orthodontic clear aligners (*n* = 1), evaluated only photographs (*n* = 2), review (*n* = 1), meeting abstracts (*n* = 1) and overlapped sample (*n* = 1). The five remaining studies [10, 15, 17, 18, 26] were included in the qualitative synthesis (Figure 1).

### 3.2 Study Characteristics

All studies (*n* = 5) [10, 15, 17, 18, 26] included in the synthesis were cross‐sectional, in Table 2, with similar methodological assessments, carried out in Saudi Arabia [18, 26], the Netherlands [15], Greece [10] and Hong Kong [17]. The number of patients included in the studies ranged from 13 to 99, aged between 12 and 28 years, but for one study [17], this was not reported, even after attempts to contact the authors. The number of evaluated teeth varied from 137 to 1653, but for one study [15], this was not reported, even after attempts to contact the authors. The time of orthodontic treatment ranged from 18 to 24 months, but this information was also not reported by two studies [10, 17], despite the attempts to contact the authors. Some studies evaluated early carious lesions involving only orthodontic brackets [10, 17, 26], and others, bands and brackets [15, 18].

**TABLE 2:** Characteristics of included studies.

Author year country	Patients (*n*) age (years) tooth (*n*)	Treatment time (months)	Orthodontic appliances	Caries evaluation	Repetition of measurements	Fluorescent method	Visual clinical method	Outcomes
Aljehani et al. [18] (2006) Saudi Arabia	13 13–17 137	24	Bands and brackets	Visual examination, DIAGNOdent and documented with a digital camera	Fluorescent method after debonding and 7 days later	DIAGNOdent	Modified Ekstrand criterion	Spearman correlation coefficient between the fluorescent and visual method was 0.40 Readings of both methods were proportional
Almosa et al. [26] (2014) Saudi Arabia	89 21.2–22.5 1653	18–24	Brackets	Visual examination and DIAGNOdent Pen	NA	DIAGNOdent Pen	ICDAS‐II modified	Spearman correlation coefficient between the fluorescent and visual method was 0.71 Correlation between methods: in tooth with ICDAS II scores 0 and 3, the reading with DIAGNOdent Pen was similar in 97% and 86% of cases In those with ICDAS II scores 1 and 2, the reading with DIAGNOdent Pen was similar in 14% and 22% of cases
Boersma et al. [15] (2005) Netherlands	62 12–18 NR	23.9–25.4	Bands and brackets	Visual examination, QLF and documented with a digital camera	Fluorescent method after debonding (T0) and 6 weeks later (T1) Visual method after debonding and 6 weeks later	QLF	Conventional visual examination	Carious lesions distribution (T1): QLF and VCE: was greater in the molars and premolars region than incisors and canines region (*p* < 0.01), especially, in the lower jaw. The patterns between both methods were similar (97%)
Kavvadia et al. [10] (2018) Greece	31 13–28 619	NR	Brackets	Direct and indirect (photos) visual examination, DIAGNOdent Pen Vista Proof	NA	DIAGNOdent Pen Vista Proof	Gorelick's criterion	**Table:** **Specificity** **Sensitivity** **Accuracy** **Direct visual method** 97% 75% 95% **Indirect visual method** 96% 64% 92% **DIAGNOdent Pen** 64% 69% 65%
Sardana et al. [17] (2022) Hong Kong	99 NR 1607	NR	Brackets	Visual examination, DIAGNOdent Pen and documented with a digital camera	Pre‐bonding Immediate post‐bonding At 6, 12 and 18‐month later	DIAGNOdent Pen	Gorelick's criterion	Correlation between methods: in the tooth with Gorelick's score 3, the DIAGNOdent Pen score had the highest mean: 19.0, compared to 0, 1 and 2: 4.56, 12.9 and 13.8**Table:** **Specificity** **Sensitivity**

Abbreviations: NR, Not reported; NA, Not applied.

Regarding DFMs, one study used DIAGNOdent (KaVo, Biberach, Germany) [18], three used DIAGNOdent Pen (KaVo, Biberach, Germany) [10, 17, 26], of these, one also used Vista Proof (Dürr Dental, AG, Munich, Germany) [10], and one used QLF (Inspektor Research Systems BV, Amsterdam, The Netherlands) [15]. For comparison, a modified Ekstrand criteria [18], an ICDAS‐II modified (original scores transformed into four: 0—healthy tooth, 1—enamel caries, 2—deep enamel caries, 3—dentine caries) [26], and Gorelick's criteria [10, 17], one used the scores: 1—early enamel caries, 2—extended enamel caries and 3—caries into dentine [10]; and the other [17], the scores: 0—no lesion, 1—slight lesion (linear shape), 2—severe lesion (band shape) and 3—cavitation, were used as VCE scores. Boersma et al. [15], performed a VCE considering as a white spot lesion the enamel surface showing a white, discoloured or cavitated region. Measurements with DFMs were performed at the pre‐bonding [17], at the time with the brackets [10, 17], repeated 6, 12 and 18 months later, at the time of debonding [10, 15, 18, 26], and repeated 7 days later [18], and 6 weeks later [15]. For indirect VCE, the measurement was performed at the time with the brackets [10]. For VCEs, these measurements were performed at the time of debonding [10, 15, 18, 26], and repeated 6 weeks thereafter [15]. Almosa et al. [26] and Kavvadia et al. [10] only performed one follow‐up measurement after debonding, either with DFM or with the VCE.

Four studies [10, 15, 17, 18] documented the entire tooth surface analysis with a digital photograph taken with the digital camera Nikon COOLPIX, Japan. The digital images were saved, printed, and used for location to guide the DFM measurements. Two studies [10, 17] also used digital photography to perform an indirect visual examination by two experienced examiners.

The Spearman correlation coefficient between DFMs and VCE scores in the studies ranged from 0.40 to 0.71 [18, 26]. Additionally Almosa et al. [26] report a clear trend that higher VCE scores are related with increase of DFM values.

Almosa et al. [26], revealed that the DFM scores were well correlated to clinical scores (ICDAS‐II modified). This correlation was assessed based on the chances of DFM being in accordance with a modified ICDAS‐II in the diagnosis of different carious lesion scores. The opposite, thus, the chances of different modified ICDAS‐II scores being in accordance with the DFM did not achieve the same correlation. Twelve of the 14 teeth classified by the VCE with the modified ICDAS‐II score 3 (dentine caries) had the same score when the DFM was used. On the other hand, of the 159 teeth diagnosed with the ICDAS‐II modified score 3 by the DFM, only 12 teeth obtained the same score when using the VCE. The results show that fluorescent methods tend to detect more positive cases, with less specificity, being more accurate when there are clear differences in the diagnosis of completely sound and dentine‐affected carious teeth [26].

In the study by Boersma et al. [15], almost all participants (97%) had one or more early enamel lesions measured by DFM and VCE. A total of 406 and 427 surfaces with caries were recorded by the QLF device, after debonding (T0) and 6 weeks later (T1), respectively, with an average loss of fluorescence for the lesions of 10.7% ± 5.8 (T0) and 10.3% ± 5.8 (T1). As for the surfaces recorded by the VCE, there were 284 (T0) and 285 (T1); an average loss of fluorescence measured by quantitative light‐induced fluorescence (QLF) method in the lesions visually detected was 12.6%, in some areas greater than 15% [15]. This corroborates the outcomes presented by another study [26].

In another study, both DIAGNOdent Pen and Vista Proof overestimated the record of early carious lesions, compared to VCE based on Gorelick's criteria (gold standard) [10]. A greater diagnostic accuracy was found for direct and indirect VCE (*p* > 0.90), compared to DFM (*p* = 0.64) [10], contrary to the other outcomes [18, 26]. Analysing the outcomes of Gorelick's scores 1 and 2, the DFM sensitivity for score 1 was low, 0.32 and 0.25, for DIAGNOdent Pen and Vista Proof, respectively, while the specificity and accuracy of both were good. For early carious lesions, the VCE method was superior to the DFM [10]. On the other hand, and corroborating with the results of previous studies [15, 18, 26], in scores 2 and 3 of Gorelick's criteria (extended carious lesions and cavitation, respectively), the DFM and VCE methods did not differ among themselves, where sensitivity and precision were higher in score 2 and 3 lesions, compared to score 1 lesions [10].

In the study by Sardana et al. [17], the values of sensitivity (78%) and specificity (82%) of DIAGNOdent Pen in delineating the presence or absence of WSLs were found to be significant (*p* < 0.001), revealing a good instrument accuracy, whereas the values of sensitivity (30%) and specificity (83%) were not significant (*p* = 0.490) for distinguishing severity of WSLs, indicating instrument failure.

### 3.3 Risk of Bias Analysis and Methodological Quality

**FIGURE 2:**
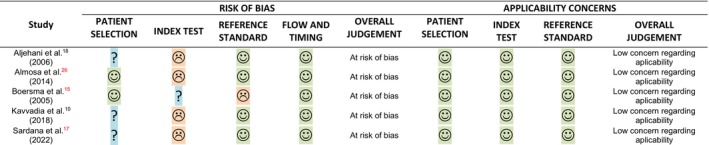
Assessment of the risk of bias in the included studies.

**FIGURE 3:**
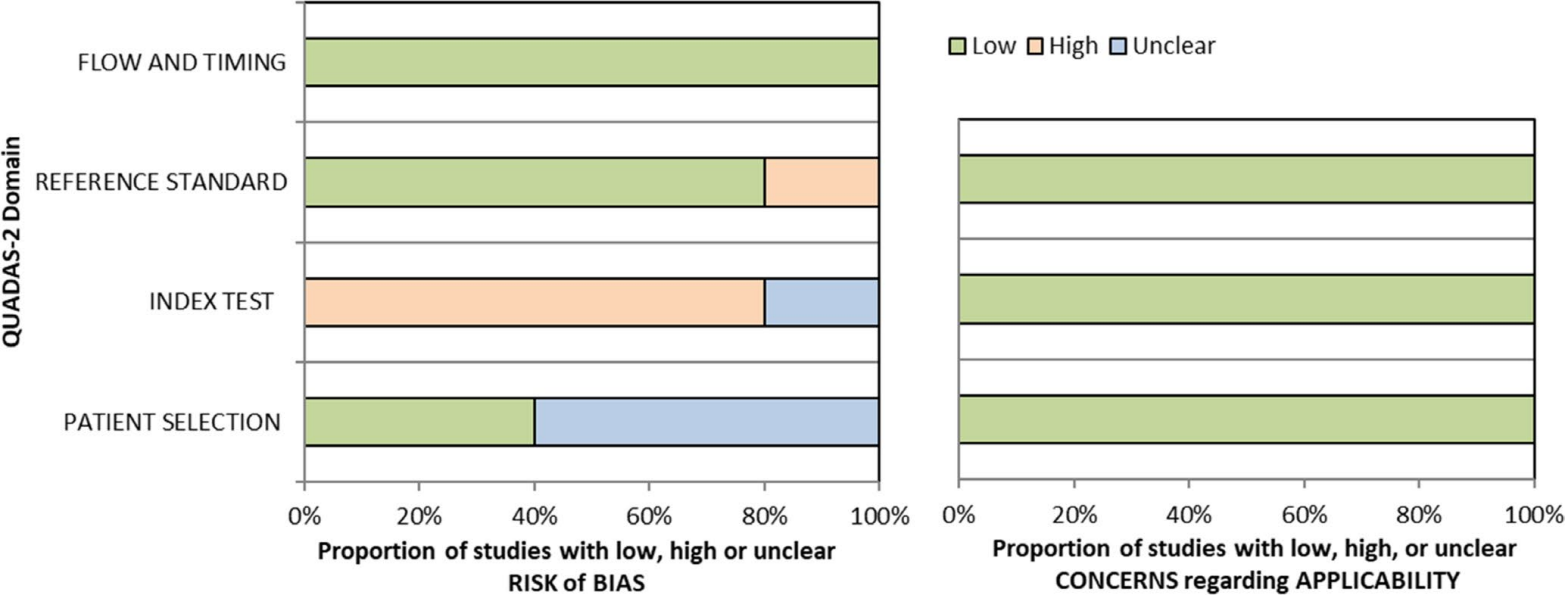
Graphical summary of study quality considering the QUADAS‐2 checklist.

In the overall judgement of risk of bias and applicability concerns [21], all studies [10, 15, 17, 18, 26] were considered ‘at risk of bias’ with ‘low concern regarding applicability’, respectively (Figure 2). Regarding each domain assessment, three studies [10, 17, 18] (60%) received ‘unclear’ scores in ‘patient selection’; four studies [10, 17, 18, 26] (80%) were scored as ‘high risk’ in ‘index text’; one study [15] (25%) was scored as ‘high risk’ in ‘reference standard’, and all studies [10, 15, 17, 18, 26] were considered at ‘low risk’ regarding study flow and timing (Figure 3) [21].

### 3.4 Certainty of Evidence

The certainty of evidence from studies in this review was ‘very low’ at the expense of insufficient data; for example, some studies made it unclear patients’ allocation methods [10, 18], number of teeth [15], not blinding at least one evaluator, and inconsistency in outcomes in Table 3.

**TABLE 3:** Certainty of evidence of correlation and sensitivity and specificity between digital fluorescent method and visual clinical examination.

Certainty assessment							Summary of findings
Number of tooth (studies)	Risk of bias	Inconsistency	Indirectness	Imprecision	Other consideration	Overall certainty of evidence	Effect
**Correlation**							
1790 (2 cross‐sectional)	Serious^a^	Not serious	Not serious	Not serious	None	⨁◯◯◯ VERY LOW	Range from 0.4 to 0.71
**Sensitivity and Specificity**							
2226 (2 cross‐sectional)	Serious^b^	Serious	Not serious	Not serious	None	⨁◯◯◯ VERY LOW	Pooled sensitivity 0.755 [0.695–0.810]* Pooled specificity 0.777 [0.758, 0.795]*

Abbreviation: CI, confidence interval.

^a^ Studies included in this analysis presented some type of risk of bias.

^b^ Study included in this analysis presented some type of risk of bias.

* Values calculated in meta‐disc software.

## 4 Discussion

Regarding DFMs for detecting carious lesions, previous systematic reviews [11, 27, 28, 29, 30, 31, 32] and meta‐analysis [14] were performed, and a few [33, 34] referred to DFMs outcomes for detection of carious lesions related to orthodontic appliances. Due to the presence of the orthodontic appliance fixed to the tooth, it was assumed that the diagnosis of carious lesions not cavitated by DFM would have an advantage in relation to the VCE, due to the maximisation of images by the attached intraoral camera [14]. However, the repetition of measurements of DFMs was performed after debonding [15, 18], pre‐bonding [17] and immediate post‐bonding [33], not sufficient to use statistical methods and combine results from two or more studies and designate a meta‐analysis [35]. Such an outcome does not invalidate the results included in this review; the opposite instigates studies with repetition of measurements before, after bonding and before debonding, in order to respond if the DFMs are ideal for detecting orthodontically carious lesions when there is the presence of metal and/or ceramic appliances fixed to the tooth.

The present systematic review differs from a narrative review since the first performs a systematic and comprehensive search, with a specific eligibility criterion, evaluation of risk of bias, synthesis of results and certainty of evidence evaluation. If used appropriately, meta‐analysis is a powerful tool for deriving meaningful conclusions. However, one should think about when a meta‐analysis could bring more confusion, or do not report consistent results instead of providing answers for the readers. Studies included in the present systematic review presented their results in different forms, with different statistical analyses and, some studies, did not present enough data to carry out a meta‐analysis (even after attempts to contact the corresponding authors). These heterogeneity limits the perform of a quantitative analysis. According to Cochrane collaboration, there are situations in which a meta‐analysis can be more of a hindrance than a help. It is important not to combine outcomes that are too diverse since in some cases consensus may be hard to reach. Combining studies that differ substantially in design and other factors can yield a meaningless summary result [36]. Additionally, meta‐analyses of studies that are at risk of bias, such as in the present systematic review, may be seriously misleading and could produce a ’wrong’ result that may be interpreted as having more credibility [36] Considering that the present systematic review is characterised by being methodologically comprehensive, transparent, and replicable and considering all these mentioned aspects, the authors decide not to combine studies results in a quantitative analysis and to only perform a detailed descriptive synthesis of the comparisons and results found in the included studies.

The results of this review showed moderate correlation between methods and that the quantification of lesion severity by determining fluorescence loss was a great benefit compared to the qualitative data obtained by VCE. In Sardana et al. [17], it was implied that DIAGNOdent Pen can correctly identify about 78% of visual WSLs around the brackets and delineate 83% correct negative results when patients do not have them. This result can be useful for clinicians in making decisions regarding the management of patients under fixed orthodontic therapy.

In the overall judgement of the risk of bias for each domain, all studies were classified as ‘at risk of bias’, due to: ‘unclear’ risk scores in domain 1 [10, 17, 18], by not reporting how their patients were selected; ‘high risk’ scores in domain 2 [10, 17, 18, 26], as the ‘index test’ was not interpreted without knowledge of the reference standard results; and a ‘high risk’ score in domain 3 [15], as the ‘reference standard’ was not a validated VCE method. Even knowing that histopathology is the gold standard for the diagnosis of carious lesions [37], validated VCE methods were considered the reference standard in this review, due to its clinical applicability. Moreover, demineralization found under histopathology might not be clinically symptomatic and relevant for clinical decision‐making [17].

A significant number of variables can adversely affect the accuracy of DFMs, such as moisture over the dental surface, the presence of biofilm, calculus or stains [14]. In this review, the selected studies were careful to control and minimise these confounding factors [10, 15, 18, 26], except for one study [17], in which patients performed their own brushing. However, as with any method involving technology, performance is also mediated in part by the examiner's experience in using the equipment and the application technique [18]. That subjectively may lead to measurement bias [16].

Visual inspection alone is adequate for most patients in daily clinical [11], but it is still a relevant challenge for clinicians, since there is a difficulty in predicting lesion volume through VCE, just by assessing the surface area [38]. Methods that accurately report the depth and volume of the lesion would be ideal for future studies, in order to evaluate the behaviour of the carious lesions, including progression or regression. Although fluorescence‐based technologies measure bacterial metabolites that penetrate the pores of the surface, they do not directly detect changes in the structure of the enamel and, therefore, are not necessarily a measurement of size and/or depth [27].

Almosa et al. [26], agree that the clinical advantages of using the DFM for the diagnosis of carious lesions are that it is objective and images can be displayed to the patient, having thus also an educational value [9]. VCE and DFM methods could be considered non‐invasive [36] and without patient discomfort. However, VCE is a subjective inexpensive method that involves little time in the clinic [18, 31], while DFM is a quantitative and objective method but with extra costs to the practice [18]. Benefits of a method with higher cost should be measured by each dentist considering their patients' profile.

## 5 Conclusions

Overall, the DFMs and the VCE present moderate correlation in the diagnosis of non‐cavitated carious lesions related to fixed orthodontic appliances, with a trend towards proportionality when the methods reached high scores. Moreover, the quantitative results and imaging possibilities may be useful in making clinical decisions about the management of patients.

## Funding

This work was supported by Coordenação de Aperfeiçoamento de Pessoal de Nível Superior.

## Conflicts of Interest

The authors declare no conflicts of interest.

## Data Availability

The data that support the findings of this study are available from the corresponding author upon reasonable request.
